# Opportunistic screening with multiphase contrast-enhanced dual-layer spectral CT for osteoblastic lesions in prostate cancer compared with bone scintigraphy

**DOI:** 10.1038/s41598-024-55427-5

**Published:** 2024-03-04

**Authors:** Ming-Cheng Liu, Chi-Chang Ho, Yen-Ting Lin, Jyh-Wen Chai, Siu-Wan Hung, Chen-Hao Wu, Jian-Ri Li, Yi-Jui Liu

**Affiliations:** 1 Department of Radiology, Taichung Veterans General Hospital, Taichung, Taiwan, ROC; 2 Ph.D. Program of Electrical and Communications Engineering, Feng Chia University, Taichung, Taiwan, ROC; 3 Institute of Clinical Medicine, National Yang-Ming University, Taipei, Taiwan, ROC; 4 Division of Urology, Department of Surgery, Taichung Veterans General Hospital, Taichung, Taiwan, ROC; 5 Department of Post-Baccalaureate Medicine, College of Medicine, National Chung Hsing University, Taichung, Taiwan, ROC; 6 Institute of Medicine, Chung Shan Medical University, Taichung, Taiwan, ROC; 7 Department of Medicine and Nursing, Hungkuang University, Taichung, Taiwan, ROC; 8 Department of Automatic Control Engineering, Feng Chia University, No. 100 Wenhwa Rd., Xitun Dist., Taichung, 407102 Taiwan, ROC

**Keywords:** Oncology, Cancer, Cancer imaging, Image processing, Metastasis

## Abstract

Our study aimed to compare bone scintigraphy and dual-layer detector spectral CT (DLCT) with multiphase contrast enhancement for the diagnosis of osteoblastic bone lesions in patients with prostate cancer. The patients with prostate cancer and osteoblastic bone lesions detected on DLCT were divided into positive bone scintigraphy group (pBS) and negative bone scintigraphy group (nBS) based on bone scintigraphy. A total of 106 patients (57 nBS and 49 pBS) was included. The parameters of each lesion were measured from DLCT including Hounsfield unit (HU), 40–140 keV monochromatic HU, effective nuclear numbers (Z_eff_), and Iodine no water (InW) value in non-contrast phase (N), the arterial phase (A), and venous phase (V). The slope of the spectral curve at 40 and 100 keV, the different values of the parameters between A and N phase (A-N), V and N phase (V-N), and hybrid prediction model with multiparameters were used to differentiate pBS from nBS. Receiver operating characteristic analysis was performed to compare the area under the curve (AUC) for differentiating the pBS group from the nBS group. The value of conventional HU values, slope, and InW in A-N and V-N, and hybrid model were significantly higher in the pBS group than in the nBS group. The hybrid model of all significant parameters had the highest AUC of 0.988, with 95.5% sensitivity and 94.6% specificity. DLCT with arterial contrast enhancement phase has the potential to serve as an opportunistic screening tool for detecting positive osteoblastic bone lesions, corresponding to those identified in bone scintigraphy.

## Introduction

Prostate cancer is the most diagnosed cancer in men in the United States, and there are an estimated 288 thousand new cases of prostate cancer annually^1^. Prostate cancer with distant metastases has a poorer prognosis. The five-year relative survival among men with prostate distant metastases is about 30 percent, compared with 100 percent for localized prostate cancer^2^. Bone is the predominant site of metastatic prostate cancer^3^. Patients with bone metastasis can eventually develop complications such as pain, pathologic fractures, or spinal cord compression. The presentation of bone metastases from prostate cancer is osteoblastic in 80% of cases^4^. However, some benign osteoblastic lesions may mimic osteoblastic bone metastases on diagnostic images^5^. This makes it challenging in diagnosing sclerotic bone lesions in a prostate cancer patient.

Bone scintigraphy using technetium-99 with methylene diphosphonate (99Tc-MDP) radionuclide is very sensitive for the detection of osteoblastic activity, providing information on osteoblastic activity, and is typically used as the first test for evaluation for suspect bone metastasis^6–8^. However, with preferential uptake of tracer at sites of active bone formation, which reflects not only the presence of neoplasm but also trauma or inflammation, the false-positivity rate of bone scintigraphy may reach up to 40%^9^. Neoplastic lesions can be distinguished from non-neoplastic lesions by conducting a thorough clinical history or correlation with other image modalities. Even though bone metastasis detection in 99Tc-MDP bone scintigraphy is not highly accurate, with a sensitivity of 71–83% and specificity of 62–87%^10,11^, it is still the most widely used method in clinical practice and is the first-line imaging modality for screening bone metastasis in patients with prostate cancer^8^.

Conventional computed tomography (CT) is another imaging tool that is widely used for staging and follow-up of metastatic prostate cancer. However, the attenuation of X-rays depends on both the material density and effective atomic number, and materials with different atomic numbers and density may have similar Hounsfield units (HU)^12^. Dual-layer detector spectral CT (DLCT) is a new type of CT study that allows material characterization beyond that possible with conventional CT. With the top layer detecting low energy spectra and the bottom layer detecting high energy spectra, DLCT provides the ability to perform two-material or multi-material decomposition through postprocessing and advanced algorithms. Among several different spectral CT mechanisms, DLCT has the advantages of low energy and the fact that high energy data are perfectly registered spatially and temporally^12^. Also, DLCT does not necessitate any additional radiation exposure and the system is always operating in a “dual-energy mode”^13^.

Although bone scintigraphy is not highly accurate in differentiating between benign and malignant uptake, it remains the first-line tool for identifying carcinomas at high risk of bone metastasis. Typically, the intensity of radioactivity accumulation is graded as high, moderate, or low relative to the uptake in ribs and the sternum^14,15^. However, bone scintigraphy is not performed for all cancer patients to detect bone metastasis; instead, CT scans are routinely conducted to evaluate metastatic lesions^16^. The areas where high radionuclide accumulation occurs are referred to as 'hot spots,' indicating abnormal uptake. The principle of bone scintigraphy relies on increased 99Tc-MDP uptake due to osteoblastic activity and local blood flow^17,18^. It is reasonable to hypothesize that the lesions exhibiting 99Tc-MDP uptake on bone scintigraphy may correspond highly to contrast-enhanced DLCT imaging.

Recently, the opportunistic or incidental use of CT data beyond the clinical indication has already demonstrated value through many studies, such as cardiovascular events^19^ and osteoporosis screening^20^. The contrast-enhanced DLCT could function as an opportunistic screening tool for osteoblastic lesions, provided it can distinguish between normal and abnormal lesions as seen in bone scintigraphy. Therefore, this study aimed to investigate the correlation between bone scintigraphy and multiphase contrast-enhanced DLCT scans related to osteoblastic lesions in prostate cancer, utilizing both arterial and venous contrast enhancement phases.

## Methods

### Study cohort

This was a retrospective case–control study conducted at a single medical center. The study was reviewed and approved by the Institutional Review Board (IRB) of Taichung Veterans General Hospital, approval number: CE23146B. The need for informed consent to participate was waived by the IRB. All methods were performed in accordance with the relevant guidelines and regulations.

From August 2021 to September 2022, patients with pathologically diagnosed prostate cancer were recruited to undergo contrast-enhanced CT on a DLCT. Basic data of the patients, including CT report, bone scintigraphy report, recent serum prostate-specific antigen (PSA) within 1 month before CT scan, and Gleason's score from a pathology report, were collected. Patients without osteoblastic bone lesions on CT image were excluded. The interval between bone scintigraphy and DLCT examination was less than 1 month.

**Figure 1:**
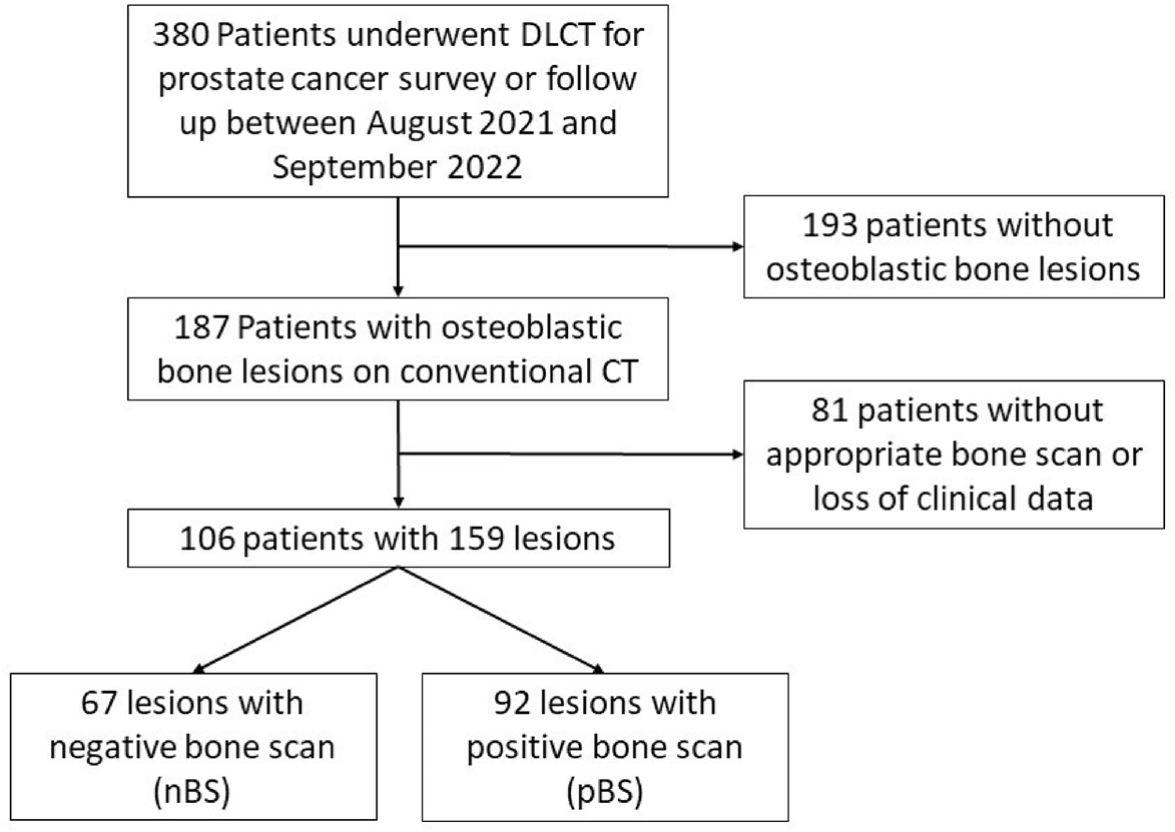
Flowchart of patient selection and analysis process.

All patients with osteoblastic lesions present in the l-spines or bilateral pelvic bones were primarily separated into two groups based on the presence of osteoblastic lesions with indeterminate morphologic features and ill-defined margins, as indicated by increased radionuclide uptake on bone scintigraphs. Osteoblastic lesions with a negative bone scintigraphy and no change in size or density within 6 months of follow-up were included in the bone scintigraphy negative group (nBS), while osteoblastic lesions with a positive bone scintigraphy or change in size or density within 6 months of follow-up were included in the bone scintigraphy positive group (pBS). Flowchart of patient selection and classification was shown on Fig. 1. The final analysis included 106 patients (mean age: 73.7 years). According to the results of bone scintigraphy, 57 patients were in the nBS group and 49 patients were in the pBS group.

### DLCT acquisition and reconstruction

All examinations were performed with a DLCT system (IQon Spectral CT, Philips Healthcare). Abdominal pelvic CT scan was performed in helical mode from diaphragm to pubic symphysis. Scanning parameters included collimation, 64 × 0.625 mm; tube voltage, 120 kVp; with automatic tube current modulation (55–177 mAs); pitch: 0.9; gantry rotation time: 0.33 s. The non-contrast CT (N phase) was acquired before the contrast agent injection. Then a 100-mL bolus of nonionic iodine contrast medium was administered IV at a rate of 3 mL/s. The arterial phase (A phase) acquisition was triggered by placing a region of interest (ROI) in the descending aorta with a trigger threshold of 150 HU, followed by the venous phase (V phase) acquisition, which was performed at 120 s after the beginning of contrast injection. The virtual monoenergetic images from 40 to 140 keV in 10-keV increments, the conventional CT images with 70 keV, the effective atomic number (Z_eff_)^21^, and the value of Iodine no water (InW)^22^ were generated from DLCT raw data. The acquired raw data were exported to a workstation (IntelliSpace Portal, version 11.1.6, Philips Healthcare), and reconstructed into axial, coronal, and sagittal images, with a slice thickness of 1 mm and an increment of 0.7 mm in the conventional polyenergetic image, and were reviewed with fixed window settings (level, 800 HU; width, 2000 HU).

### Image analysis

Two independent readers (reader 1 with 10 years of experience in genitourinary and abdomen radiology; reader 2 with 3 years of experience in radiology) drew a single circular ROI according to the osteoblastic lesions visible on the N phase images, and up to 3 ROIs were chosen per patient. The average value measured by the two independent readers was considered to be the final value included in the statistical results. Each ROI was 50 ± 5 mm^2^ and was placed at the center of the visible bone lesion. For the nBS group, the ROIs were placed at the most sclerotic portion within the normal bone boundary, and exophytic osteophytes were excluded. Using the copy-and-paste function, the ROIs on the N phase were copied to the A phase and V phase images.

For each ROI for the lesions, the HU values of conventional CT images, and the virtual monoenergetic images, the values of Z_eff_ and InW were recorded, and the value of InW of the largest artery at the same craniocaudal level (abdominal aorta at the abdominal level, common iliac artery or external iliac artery at pelvic level) were collected. To mitigate the bias of InW calculation and CT scan, the InW values were further normalized by dividing the lesion InW by the InW of the largest artery (normalized InW = InW_lesion_/InW_artery_). The normalized InW was denoted as nAInW for the artery phase and nVInW for the venous phase. For further calculation, the HU values on virtual monoenergetic image sets from 40 to 140 keV were used to generate spectral curves, which were simplified into a slope of the spectral curve at 40 keV and 100 keV (slope = (HU40–HU100)/60)^23,24^. The aforementioned values were obtained by measuring ROIs on the images captured during the N phase, A phase, and V phase, respectively.

The contrast enhancement contributions of different phases in DLCT were evaluated in this study. The enhanced HU among different phases, including A phase to N phase ((A-N)HU), V phase to N phase ((V-N)HU), and V phase to A phase ((V-A)HU), were calculated to quantify the enhancement and enhancement pattern. Similarly, the enhanced InW among different phases, including A phase to N phase ((A-N)InW), V phase to N phase ((V-N)InW), and V phase to A phase ((V-A)InW), were also calculated. In addition, the normalized InW between phases to N phase were defined as: (A-N)InW% = (A-N)InW_lesion_/(A-N)InW_artery_x100%, and (V-N)InW% = (V-N)InW_lesion_/(V-N)InW_artery_x100%.

### Statistical analysis

All data were analyzed with SPSS software (version 23.0, IBM). The Kolmogorov–Smirnov test was performed to assess the normality of distribution for quantitative data. The categorical variables were compared using Fisher’s exact test. The parameters of the two groups were compared using Mann–Whitney *U* tests for continuous data, and are expressed as mean ± SD.

Interreader agreement between the two readers was assessed using the intraclass correlation coefficient (ICC) and 95% CIs, based on an absolute agreement. ICC values of less than 0.5, 0.5–0.75, 0.75–0.9, and greater than 0.90 were considered to indicate poor, moderate, good, and excellent reliability, respectively^25^.

The area under the curve (AUC) was computed using receiver operator characteristic (ROC) analysis to assess the discriminatory performance of CT parameters in differentiating the pBS group from the nBS group. Only the parameters that exhibited significant differences between the two groups, as determined by Mann–Whitney *U* tests, were included in the AUC calculation. Furthermore, the significant parameters in the Mann–Whitney *U* tests were selected and combined to generate a hybrid predicting model using logistic regression analysis for differentiating the pBS group from the nBS group^26^. The sensitivity and specificity of each parameter were calculated, and the optimal cut-off value for each parameter was determined using the Youden index. A value of p < 0.05 was considered statistically significant. Finally, the DeLong test of AUCs was used to determine the significant difference among the methods for differentiating between the pBS group and nBS group.

## Results

### Patients’ characteristics

The patients’ basic data are shown in Table 1. Patients in the bone scintigraphy positive group (pBS) had significantly higher recent serum prostate-specific antigen (PSA) (37.81 ± 154.13 ng/mL vs. 638.04 ± 1367.49 ng/mL) and higher Gleason's score (8.58 ± 0.94 vs. 8.02 ± 1.05).

**Table 1:** Comparison of basic characteristics between the nBS group and the pBS group.

	Total (n = 106)		nBS (n = 57)		pBS (n = 49)		*p* value
	Median	IQR	Median	IQR	Median	IQR	
Age	72.00	67.75–80	74.00	69.5–79.5	71.00	66–80.5	0.180
Gleason’s score	8.00	7–9	8.00	7–9	9.00	8–9	0.005
Recent PSA*	8.40	1.62–100.44	1.91	0.27–9.84	84.24	9.57–942.05	< 0.001

*nBS* negative bone scintigraphy, *pBS* positive bone scintigraphy, *PSA* prostate-specific antigen.

*Assessed within 1 month by the time abdominal CT was performed.

### Interreader agreement on conventional CT and DLCT parameters

The interreader agreement of conventional CT and DLCT parameters was excellent at the single phase (N phase, A phase and V phase: ICC, 0.95–0.98), moderate to good for subtracting values (A phase–N phase, V phase–N phase, and V phase–A phase: ICC, 0.52–0.86), with only the HU value of V phase–N phase showing poor agreement (ICC, 0.49).

### Virtual monoenergetic images (VMIs)

Among those patients, 159 ROIs were measured. Hounsfield units of virtual monoenergetic image from 40 to 140 keV in 10-keV increments were measured at the N phase, A phase, and V phase (Supplementary file). None of the virtual monoenergetic images revealed significant differences between the two groups.

### Other spectral parameters

Table 2 summarizes all other spectral parameters in the two groups. The absolute values of conventional HU, Z _eff_, as well as Iodine no water and slope of spectral curve at N phase, A phase, and V phase showed no significant differences between the two groups. The normalized Iodine no water values at A phase and V phase still showed no significant differences between the two groups. However, as shown in Table 2, the bone scintigraphy negative (nBS) group showed significantly less enhancement than the pBS group on both A phase (6.01 ± 13.93HU vs. 40.99 ± 26.33HU, p < 0.001) and V phase (9.49 ± 15.81HU vs 42.56 ± 32.44HU, p < 0.001), as compared with the non-contrast phase. This phenomenon was not only present in the HU values of conventional CT, but was also observed in the iodine non-water, and slope of the spectral curve. The subtracting values of: Iodine no water in A phase–N phase (0.61 ± 0.46 mg/ml vs. 1.94 ± 0.78 mg/ml, p < 0.001), Iodine no water in V phase–N phase (0.85 ± 0.47 mg/ml vs. 2.02 ± 0.97 mg/ml, p < 0.001), slope of spectral curve in A phase–N phase (0.95 ± 0.77 vs. 2.35 ± 0.95, p < 0.001), and slope of spectral curve in V phase–N phase (1.18 ± 0.63 vs. 2.44 ± 1.17, p < 0.001), were significantly lower in the nBS group than in the pBS group. Moreover, the subtracting values of all parameters in V phase–A phase revealed no significant differences between the two groups.

**Table 2:** Different phase and parameter of dual-layer spectral detector CT images of the nBS group and the pBS group.

	nBS (n = 67)		pBS (n = 92)		*p* value
	Mean	± SD	Mean	± SD	
N (HU)	513.47	± 231.97	510.27	± 195.50	0.702
A (HU)	519.48	± 230.96	551.26	± 192.21	0.144
V (HU)	522.96	± 230.21	552.83	± 196.01	0.172
N (zeff)	10.59	± 0.89	10.51	± 0.79	0.656
A (zeff)	10.77	± 0.86	10.96	± 0.70	0.088
V (zeff)	10.81	± 0.84	10.95	± 0.76	0.230
N (InW)	10.72	± 4.84	10.23	± 4.18	0.736
A (InW)	11.33	± 4.84	12.17	± 4.20	0.094
nAInW	1.04	± 0.51	1.14	± 0.43	0.065
V (InW)	11.57	± 4.89	12.25	± 4.46	0.171
nVInW	3.08	± 1.40	3.13	± 1.18	0.519
N (slope)	13.21	± 5.95	12.69	± 5.17	0.813
A (slope)	14.15	± 6.09	15.04	± 5.17	0.135
V (slope)	14.38	± 6.06	15.14	± 5.50	0.223
A-N (HU)	6.01	± 13.93	40.99	± 26.33	< 0.001*
V-A (HU)	3.47	± 10.54	1.57	± 18.74	0.074
V–N (HU)	9.49	± 15.81	42.56	± 32.44	< 0.001*
A-N (InW)	0.61	± 0.46	1.94	± 0.78	< 0.001*
A-N (InW)%	5.48	± 4.18	17.81	± 6.16	< 0.001*
V-A (InW)	0.24	± 0.45	0.08	± 0.73	0.051
V–N (InW)	0.85	± 0.47	2.02	± 0.97	< 0.001*
V–N (InW)%	22.28	± 12.50	50.91	± 23.70	< 0.001*
A-N (slope)	0.95	± 0.77	2.35	± 0.95	< 0.001*
V-A (slope)	0.23	± 0.70	0.09	± 0.88	0.095
V–N (slope)	1.18	± 0.63	2.44	± 1.17	< 0.001*

nAInW and nVInW = normalized iodine no water = Lesion InW/ Large artery InW. slope (HU40 keV-HU100 keV)/60.

*HU* hounsfield unit from conventional CT images, *Zeff* effective atomic number, *InW* iodine no water (mg/ml).

*Significant difference.

### Diagnostic performance of each parameter

**Figure 2:**
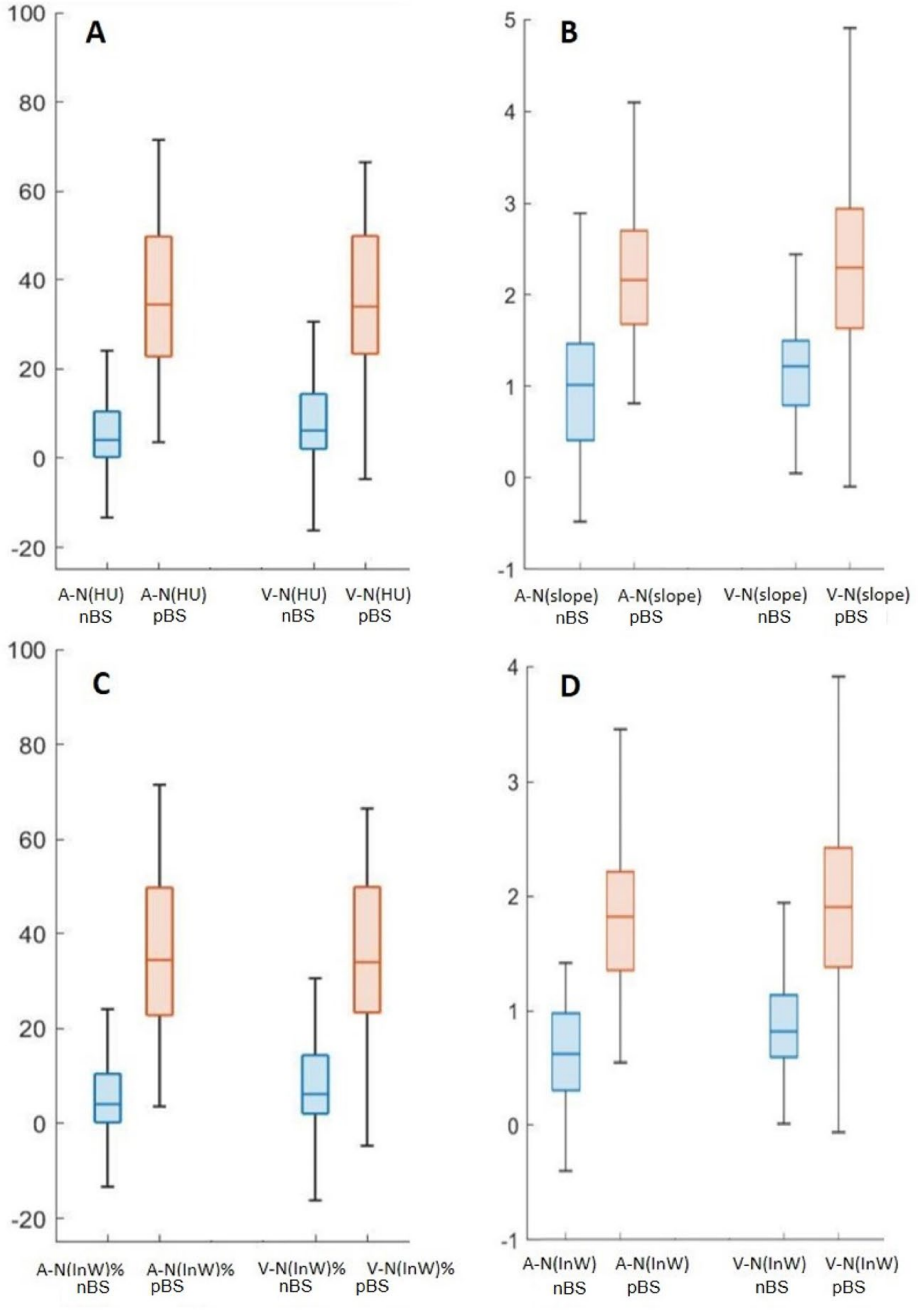
Box-plot between nBS group and pBS group and A-N and V-N data for HU, slope, InW, and (InW)%. ***p < 0.001.

Figure 2 shows the box-plot between pBS and nBS groups for the parameters with a significant difference, and Table 3 shows the diagnostic performance of those parameters for differentiating the pBS group and the nBS group. For a single factor differentiating the pBS group from the nBS group, the subtracting value of normalized InW in the arterial phase ((A-N)InW%) had the highest AUC of 0.967, followed by the subtracting value of InW in the arterial phase ((A-N)InW), which had an AUC of 0.955, and the subtracting value of HU in the arterial phase ((A-N)HU)) had an AUC of 0.939. In the hybrid predicting model, combining all parameters with a significant difference between nBS and pBS yielded the best model with an AUC of 0.988. In addition, combining all parameters of A-N with a significant difference between nBS and pBS resulted in an AUC of 0.986, while combining all parameters of V-N with a significant difference between nBS and pBS had an AUC of 0.925.

**Table 3:** Area under the curve (AUC), sensitivity and specificity of the different parameters in the subtraction data for distinguishing the pBS group and the nBS group.

	AUC	Sensitivity (%)	Specificity (%)	Optimal threshold	ROC p value
Hybrid all	0.988	95.5	94.6	0.45	
Hybrid all A-N	0.986	94	96.7	0.51	0.004*
Hybrid all V-N	0.925	88.1	88	0.49	
A-N (HU)	0.939	92.4	86.6	14.5	0.003*
V-N (HU)	0.885	83.7	83.6	16.3	
A-N (InW)	0.955	84.8	92.5	1.28	0.007*
V-N (InW)	0.891	72.8	92.5	1.18	
A-N (InW)%	0.967	95.7	86.6	10.09	0.005*
V-N (InW)%	0.895	83.7	86.6	34.54	
A-N (slope)	0.889	76.1	86.6	1.34	0.233
V-N (slope)	0.853	69.6	91.0	1.45	

Hybrid All = a logistic regression model using a combination of A-N(HU), A-N(InW), A-N(InW)%, A-N(slope),V-N(HU), V-N(InW), V-N(InW)%, and V-N(slope).

Hybrid All A-N = a logistic regression model using a combination of A-N(HU), A-N(InW), A-N(InW)%, and A-N(slope).

Hybrid All V-N = a logistic regression model using a combination of V-N(HU), V-N(InW), V-N(InW)%, and V-N(slope).

*Significant difference.

**Figure 3:**
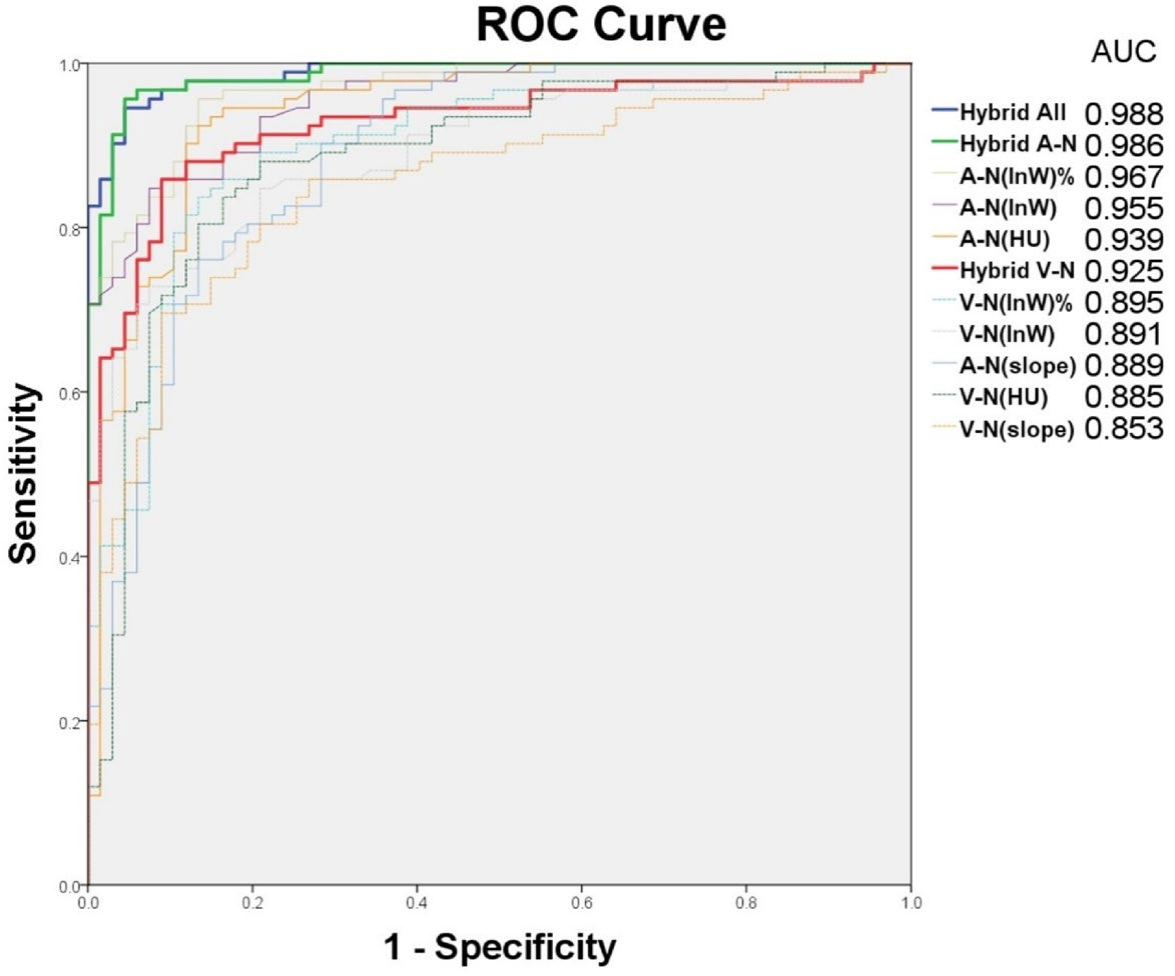
Graph shows ROC curves of dual-layer spectral detector CT parameters for differentiation of pBS group and nBS group. Depicted parameters for arterial phase or venous phase enhancement ranging from 0.853 to 0.988.

**Figure 4:**
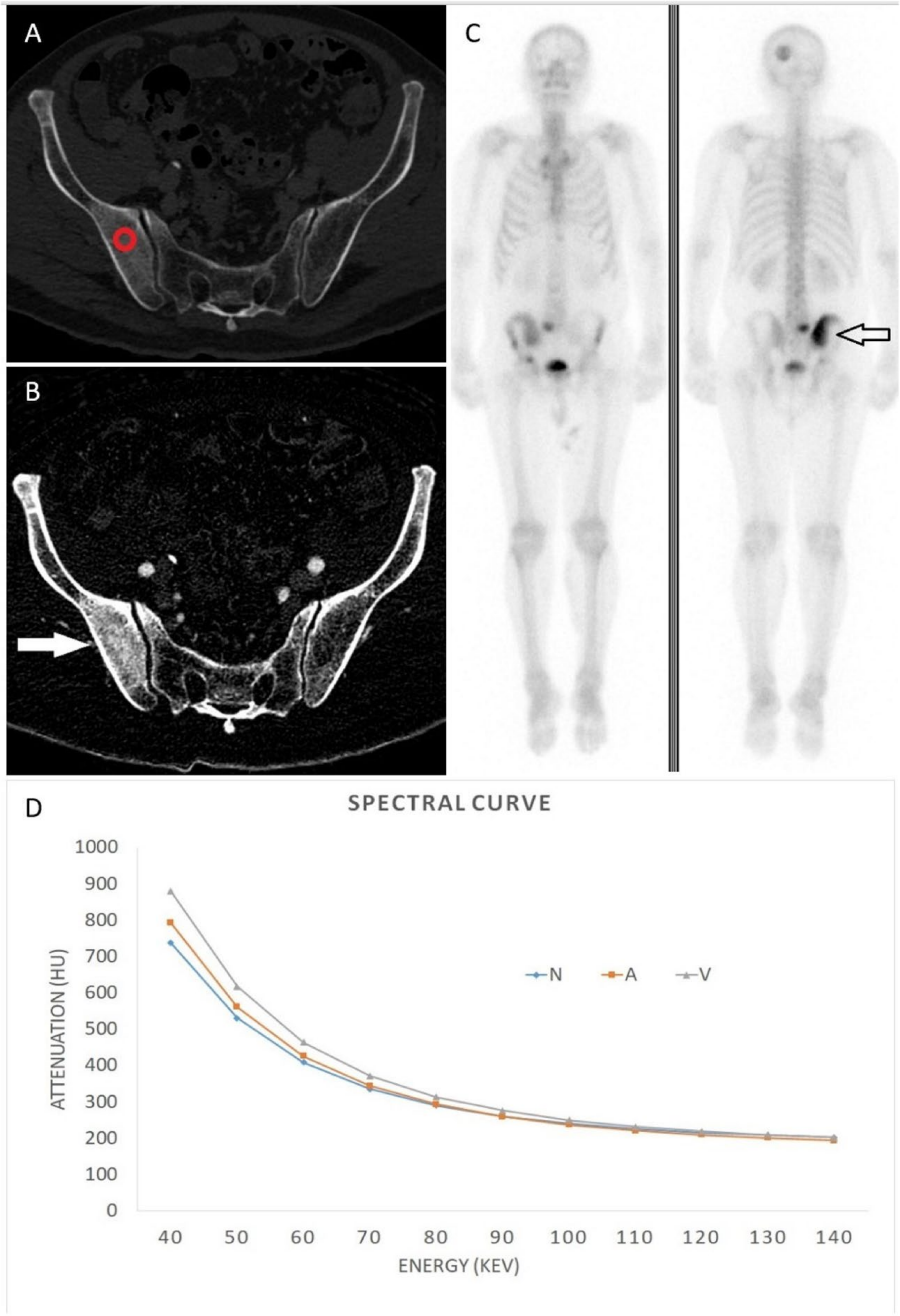
A 65-year-old male diagnosed with prostate cancer exhibiting positive findings on the bone scintigraphy at right iliac bone. (**A**) Conventional CT image shows osteoblastic lesion at right iliac bone abutting sacroiliac joint. (**B**) Iodine no water image of arterial phase, the red circle represents the ROI measured. The iodine no water value is 7.46 mg/ml. Compared with non-contrast phase, the arterial phase enhancement is 0.74 mg/ml and the venous phase enhancement is 1.61 mg/ml. (**C**) Bone scintigraphy shows increased MDP uptake at left parietal bone, the sacrum, the right iliac bone, and the right acetabulum, demonstrating multiple bone metastasis. (**D**) Spectral curve of different phases revealed separation at low energy. *N* non-contrast, *A* arterial phase, *V* venous phase.

**Figure 5:**
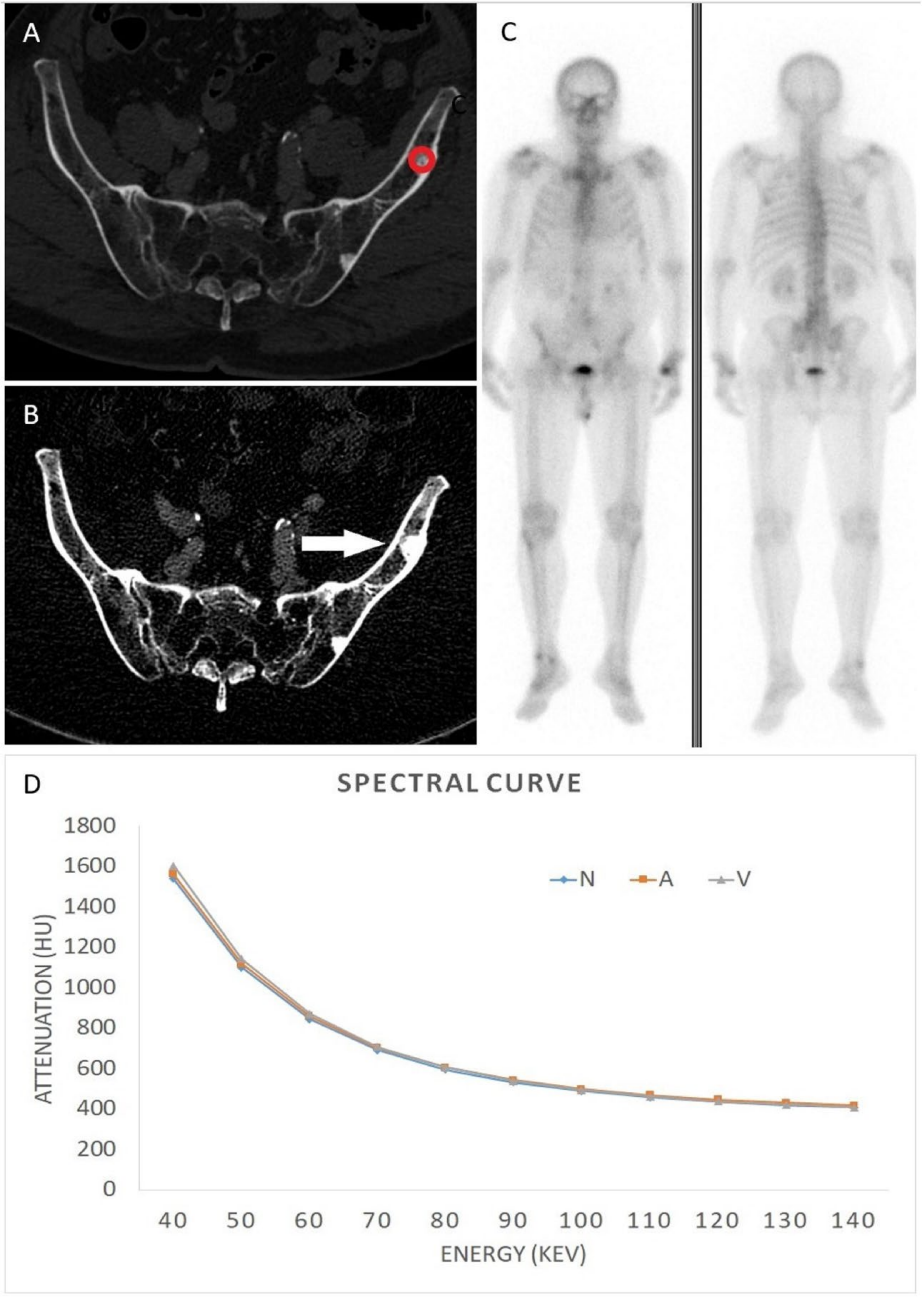
A 70-year-old male diagnosed with prostate cancer exhibiting negative findings on the bone scintigraphy at left iliac bone. (**A**) Conventional CT image shows two osteoblastic nodular lesions at left iliac bone. (**B**) Iodine no water image of arterial phase, the red circle represents the ROI measured. The iodine no water value is 14.3 mg/ml. Compared with non-contrast phase, the arterial phase enhancement is 0.14 mg/ml and the venous phase enhancement is 0.8 mg/ml. (**C**) Bone scintigraphy shows no abnormal MDP uptake at bilateral iliac bone. (**D**) Spectral curve of different phases revealed no separation at any energy. *N* non-contrast, *A* arterial phase, *V* venous phase.

There were significantly higher AUC values for the hybrid predicting model, HU, InW, InW% and slope at A phase–N phase than at the V phase–N phase, with p-values of approximately 0.004, 0.003, 0.007, 0.005, and 0.233, respectively (Table 3). Figure 3 shows the ROC curve of all parameters with significant differences between the nBS group and the pBS group. Figures 4 and 5 depict the conventional and DLCT image features of the pBS group and the nBS group.

## Discussion

In this study, our aim was to assess whether DLCT with multiphase contrast enhancement could serve as an opportunistic screening tool for detecting osteoblastic lesions in patients with prostate cancer, in comparison to bone scintigraphy. Our results showed that the performances of all single factors in the arterial phase were higher than those in the venous phase (Table 3). The best performance was observed with the hybrid model incorporating all significant parameters, which achieved an AUC of 0.988, 95.5% sensitivity, and 94.6% specificity. These findings indicate that DLCT with multiphase contrast enhancement performs similarly to bone scintigraphy in detecting osteoblastic lesions in patients with prostate cancer.

The clinical significance of bone metastasis is that it not only determines the staging of prostate cancer, but also influences the treatment strategy and prognosis^27^. For low risk localized prostate cancer, conservative management with active surveillance of PSA or imaging studies could be considered. For localized prostate cancer patients with longer life expectancy, radical prostatectomy, external beam radiation, brachytherapy or cryotherapy are the typical treatments of choice. For advanced prostate cancer, systemic treatment, including hormone therapy, chemotherapy, immunotherapy, or Radium-223 therapy, is suggested, and more prominent systemic adverse effects with poorer prognosis is inevitable^28^.

Bone scintigraphy with 99Tc-MDP is the first-line imaging modality and is a widely used method worldwide for the screening of bone metastasis in patients with prostate cancer^8^, regardless of false-positivity due to traumatic, infectious, or inflammatory disease^9^. Although some image modalities, such as MRI^29^, PET/CT^29^, and SPECT^29^, achieve a higher accuracy in bone metastasis detection, bone scintigraphy with 99Tc-MDP is still suggested as the first-line imaging modality because the other modalities are not available for whole body scan or for use as the first strategy in clinical practice^30^. Our results demonstrated the ability of DLCT with dynamic phase contrast enhancement in detecting abnormal osteoblastic bone lesion was equal to bone scintigraphy. The possible reason is that the lesions with highly increased uptake in bone scintigraphy are correlated with bone turnover and bone perfusion, whereas CT with dynamic phase contrast enhancement is also related to bone activity and blood flow. Hence, DLCT with contrast enhancement may be a potential tool for an opportunistic screening in bone metastasis detection.

A previous study demonstrated some utilities of non-contrast spectral CT for diagnosing bone metastatic lesions from prostate cancer^16^. Another study revealed an improvement in the differential diagnosis of osteoblastic metastasis from bone island with non-contrast spectral CT^24^. There is also research on post-contrast iodine density on spectral CT for diagnosis of vertebral bone metastasis^31^. Prior studies demonstrated the determination of prevalent bone metastases based on iodine density measures in comparison to soft tissue organs was found to be difficult due to (a) similar characteristics of calcium and iodine in dual-energy CT^32^, aggravated by (b) the high intra-individual variance of bone mineral content, (c) the contrast between trabecular bone and metastases, and (d) partial volume effects of trabecular^31^. Our data confirm these results. In our study, we found that no parameter demonstrated a significant difference between the nBS group and the pBS group in each single phase of DLCT. The study also revealed that age and volumetric bone mineral density (vBMD) interfered with iodine density in vertebral bone^31^. Thus, it is hard to differentiate between lesions with a single phase of iodine density. However, to the best of our knowledge, no previous research has explored the utilization of different time phases of contrast-enhanced spectral CT for distinguishing osteoblastic bone lesions in prostate cancer correlated with bone scintigraphy.

The angiogenesis feature of bone metastasis tumor creates a vascular network, replacing normal bone vasculature with a disordered network of tortuous arteries winding throughout the bone^33^. Our study demonstrated that, by adding 3 phases into the calculation, the pBS group revealed significant differences in both the arterial phase and venous phase of enhancement. Nonetheless, no significant tendency of delayed enhancement or washout pattern was observed. Based on the Youden Index from ROC analysis, arterial enhancement demonstrated better performance than venous enhancement. The arterial enhancing proportion of Iodine no water [A-N(InW)%] showed the highest AUC of 0.97, yielding a sensitivity of 95.7% and a specificity of 86.6%. That is, the dual-layer spectral detector CT with dynamic contrast enhancement can achieve similar differentiation results as those achieved using bone scintigraphy and can also perform quantitative analysis of osteoblastic lesions. The subtracting value with an emphasis on the enhancing portion can provide a more differentiated value for osteoblastic bone lesions in patients with prostate cancer. Furthermore, the parameters obtained at the arterial phase provided greater diagnostic accuracy than the values obtained during the venous phase. Among the DLCT parameters for differentiation, the value of iodine no water was more useful than the value of mono E slope, while Zeff was not helpful in differentiation.

In our research, we observed the highest correlation between the arterial phase of DLCT imaging and bone scintigraphy. This may be related to the rapid bone turnover rate within the bone microenvironment when 99Tc-MDP uptake in the bone scintigraphy, occurring simultaneously with heightened perfusion during the arterial phase in the DLCT. In the imaging assessment of prostate cancer staging, both bone scintigraphy and contrast-enhanced CT are equally important. These findings suggest that in the future, multiphase contrast-enhanced DLCT could potentially serve as an opportunistic screening tool for evaluating osteoblastic bone lesions within a single CT examination. This substitution could reduce the additional radiation dose associated with performing bone scintigraphy while retaining the advantages of DLCT in assessing soft tissue or pulmonary metastatic lesions.

Our study demonstrated that the capability of multiphase contrast-enhanced DLCT is equivalent to bone scintigraphy in identifying bone lesions with significantly increased uptake. However, both modalities have their advantages and limitations. While bone scintigraphy provides a whole-body scan, its focus is primarily on bone lesions. The advantage of bone scintigraphy is that it allows observation of the entire skeletal system in a single examination, including the axial skeleton and all regions of the limb bones. In contrast, CT scans can only assess the condition within the scanned area, and regions not scanned cannot be evaluated. Conversely, CT scans cover local body areas and various organs. Apart from bones, which are the most common metastatic sites for prostate cancer, distant lymph nodes, the liver, or the thorax are also frequent sites for distant metastasis^34^. CT scans with contrast medium administration are routinely recommended for the clinical staging of prostate cancer.

This study has several limitations. First, this was a retrospective investigation and the data were collected after the confirmation of prostate cancer diagnosis, which introduces possible selection bias and missing cases (i.e. underdiagnosis). Secondly, the pBS group was not confirmed histologically, in accordance with institutional practice. The sensitivity and specificity of whole body bone scintigraphy was 71–83% and 62–87%, respectively^10,11^, indicating the possibility of false positive or false negative findings on both bone scintigraphy and DLCT. Future prospective studies could investigate the potential discordance between the results of whole body bone scintigraphy and the parameter findings of DLCT. Semi-quantitative or quantitative information can be obtained on bone scintigraphy through the maximum lesion to normal bone count ratio (ROImax) or computer-aided design assessment^35,36^. However, in our institution, all assessments of bone scintigraphy rely solely on visual interpretation to derive qualitative results, which represents a limitation in our research. Third, the sample size was relatively small, so future investigations should include more cases. Future studies should investigate the correlation between DLCT images and MRI images, PET/CT, and histological results.

## Conclusion

DLCT with dynamic contrast enhancement has the potential to opportunistically screen and detect osteoblastic lesions in patients with prostate cancer due to its excellent correlation performance with bone scintigraphy in differentiating osteoblastic lesions. It may be considered that DLCT with arterial contrast enhancement phase be routinely included as part of the initial screening for prostate cancer. This imaging modality can assist in distinguishing the nature of osteoblastic lesions.

### Supplementary Information

Supplementary Information.

## Data Availability

The datasets used or analyzed during the current study are available from the corresponding author on reasonable request.

## Abbreviations

DLCT: Dual-layer detector spectral CT
nBS: Negative bone scintigraphy
pBS: Positive bone scintigraphy
N phase: Non contrast CT
A phase: Arterial phase CT
N phase: Venous phase CT
Z_eff_: Effective atomic number value
MonoE: Virtual mono-energetic images
InW: Iodine no water value
nAInW: Normalized iodine no water value in arterial phase
nVInW: Normalized iodine no water value in venous phase
